# Incremental 2D self-labelling for effective 3D medical volume segmentation with minimal annotations

**DOI:** 10.1186/s12880-025-01991-9

**Published:** 2025-11-07

**Authors:** Matthew Anderson, Maged Habib, David H. Steel, Boguslaw Obara

**Affiliations:** 1 School of Computing, Newcastle University, 1, Urban Sciences Building, Science Square, 1 Science Square, Newcastle Upon Tyne, NE4 5TG UK; 2 Sunderland Eye Infirmary, National Health Service, Queen Alexandra Rd, Sunderland, SR2 9HP UK; 3 Biosciences Institute, Newcastle University, Catherine Cookson Building, Newcastle Upon Tyne, NE2 4HH UK

**Keywords:** Deep learning, Minimal annotations, Medical image segmentation, Self-labelling

## Abstract

**Background:**

The development and application of deep learning-based models have seen significant success in medical image segmentation, transforming diagnostic and treatment processes. However, these advancements often rely on large, fully annotated datasets, which are challenging to obtain due to the labour-intensive and costly nature of expert annotation. Therefore, we sought to explore the feasibility and efficacy of training 2D models under severe annotation constraints, aiming to optimise segmentation performance while minimising annotation costs.

**Methods:**

We propose an incremental 2D self-labelling framework for segmenting 3D medical volumes from a single annotated slice per volume. A 2D U-Net is first trained on these central slices. The model then iteratively generates and filters pseudo-labels for adjacent slices, progressively fine-tuning itself on an expanding dataset. This process is repeated until the entire training set is pseudo-labelled to produce the final model.

**Results:**

On brain MRI and liver CECT datasets, our self-labelling approach improved segmentation performance compared to using only the sparse ground-truth data, increasing the Dice Similarity Coefficient and Intersection over Union by up to 15.95% and 26.75%, respectively. It also improved 3D continuity, reducing the 95th percentile Hausdorff Distance from 69.88 mm to 36.46 mm. Parameter analysis revealed that a gradual propagation of high-confidence pseudo-labels was most effective.

**Conclusion:**

Our framework demonstrates that a computationally efficient 2D model can be leveraged through self-labelling to achieve robust 3D segmentation performance and coherence from extremely sparse annotations, offering a viable solution to reduce the annotation burden in medical imaging.

## Introduction

Image segmentation falls within the domain of computer vision and digital image processing. The objective of image segmentation is to categorise comparable regions or segments within an image based on their respective class labels. This technique has been utilised in a wide range of applications such as robot vision [1], agricultural monitoring [2], and facial recognition [3]. A particularly impactful application has been medical image segmentation, where the goal is to identify and outline specific anatomical structures, such as organs and tumours, within medical images. This fundamental step is integral to various clinical applications, including computer-aided diagnosis [4], treatment planning [5], and radiation therapy [6]. Precise segmentation yields dependable volumetric and shape information, facilitating subsequent clinical tasks such as disease diagnosis, treatment guidance, and response prediction.

Classical image segmentation, widely researched for decades, largely falls into three categories: boundary-based, region-based, and hybrid-based segmentation [7]. However, classical techniques can struggle when dealing with diverse and complex image content. AI-powered machine learning algorithms, particularly those employing deep learning frameworks, have demonstrated a remarkable capacity to learn intricate patterns and features within datasets. This proficiency equips them to effectively manage increasingly complex image data. In particular, the introduction of U-Net [8] has led to substantial advances in medical image segmentation tasks. However, training these deep learning models is challenging, as it often requires a large amount of accurately annotated data, a time-consuming and costly process for clinical experts [9]. Conversely, anonymised unlabelled medical data is often readily available, and effectively harnessing this resource has the potential to improve segmentation performance at a minimal cost. Various techniques have been introduced to tackle this problem, including a range of semi-supervised and weakly-supervised learning approaches [10–14]. Pseudo-labelling [15, 16] is a type of semi-supervised learning whereby pseudo-labels are created using predictions from a trained model. These pseudo-labelled data points are then included in the training set to improve model performance, and this process is repeated iteratively in a self-training cycle.

Incremental learning involves acquiring knowledge or skills step by step while building on existing knowledge and is a key feature of natural intelligence [17]. It is characterised by the gradual accumulation of information, allowing systems to refine their understanding of tasks and concepts over time. In particular, humans exhibit remarkable learning capabilities in dynamic environments. Through extensive neurophysiological evolution, the brain has developed the capacity to gradually accumulate and retain knowledge on successive sequential tasks [18]. Consequently, the principles governing knowledge processing in the brain, explored through biological methodologies, inspire the advancement of computational approaches. This area of machine learning research has been rapidly expanding, fuelled by the potential utility of deploying continual learning algorithms for applications such as recommender systems [19], autonomous driving [20] and aid in investment decisions [21].

This study considers a scenario where a medical image dataset comes with extremely minimal annotations. This situation is not uncommon given the scarcity of large volumes of expertly annotated medical image data, which can hinder the growth of deep learning in medical image diagnosis [22]. We investigate how using only a central-annotated slice from each training volume, we can slowly propagate pseudo-labels across the entire volume in an iterative self-labelling approach using a 2D deep learning segmentation model. Our exploration is rooted in the principle of building upon familiar information incrementally. Beginning with a small amount of ground-truth data, analogous to foundational knowledge, we aim to emulate a learning trajectory where understanding builds upon familiar concepts. This strategy responds to the challenges posed by the limited availability of annotated data in medical imaging. By gradually exposing the model to variations of data it has already encountered, we anticipate that this measured approach may unlock novel avenues for enhancing model performance in medical volume segmentation.

Experimental results with the proposed self-labelling method on multiple diverse datasets show that the approach is superior to that of a model using only a small amount of ground-truth annotated data. Our approach achieves substantial improvements across multiple segmentation metrics, with the Dice Similarity Coefficient DSC and Intersection over Union IoU increasing by an average of 15.95% and 26.75% respectively over the baseline, highlighting the potential to alleviate annotation constraints for medical volumes. The main contributions are:

- We introduce a simple and computationally efficient 2D self-labelling framework to improve 3D medical volume segmentation from extremely sparse annotations (Sect. 4).
- We demonstrate through comprehensive analysis that the incremental propagation of pseudo-labels significantly improves 3D segmentation continuity and boundary accuracy (HD95) from a single annotated slice (Sect. 5).

The remainder of this paper is structured as follows. We begin by reviewing related works in pseudo-labelling and semi-supervised learning in Sect. 2. Section 3 then details the datasets and model architectures used in our experiments. In Sect. 4, we present our incremental self-labelling framework and training methodology. We report our quantitative and qualitative results in Sect. 5, followed by a comprehensive discussion of their implications and limitations in Sect. 6. Finally, Sect. 7 concludes the paper and suggests directions for future research.

## Related work

Our research is closely aligned with the general application of pseudo-labeling which has been an active area of research in semi-supervised learning over recent years [15, 23]. Consistency regularisation is a commonly employed technique in pseudo-label learning [24]. This method compels the model to produce consistent predictions for inputs subjected to varying degrees of perturbation, such as weakly and strongly augmented images. Another prevalent approach is the Mean Teacher framework [25], which operates as a teacher-student system. To address the issue of confirmation bias [26], where incorrect pseudo-labels may impact the model, some existing methods suggest refining predictions considering their confidence scores [27, 28], where only predictions with high confidence are utilised as pseudo-labels, while ambiguous ones are disregarded.

Cai et al. [29] introduced a notably relevant approach, the weakly supervised slice propagated segmentation (WSSS) method, using convolutional neural networks (CNNs) for the segmentation of volumetric lesion in CT images. The WSSS method utilises response evaluation criteria in solid tumours (RECIST) annotations as weak supervision, generating initial lesion segmentation on RECIST-marked slices, and extending the segmentation to achieve volumetric completeness. Addressing the challenges associated with manual 3D segmentation, this method shows performance comparable to that of fully supervised counterparts on annotated datasets. Bitarafan et al. [30] addressed segmentation from a single annotated 2D slice per volume using a self-training method named 3D-SegReg. Their framework is built on a hybrid architecture that iteratively combines a 3D segmentation network (3D-SegNet) with a separate 2D registration network (2D-RegNet). In their process, the 2D network propagates labels to adjacent slices via registration, and these predictions are then aggregated with the output of the main 3D network to generate pseudo-labels. By fusing 2D inter-slice registration with 3D volumetric processing, their work demonstrates a powerful method for learning from sparse annotations.

Despite the abundance of research on 3D AI segmentation techniques, strategies for enhancing 3D segmentation performance using solely 2D AI models with sparse annotations remain a less-explored area. The potential advantages of 2D models, namely their significantly reduced computational demands, make them a compelling but underutilised option in this context. This paper aims to fill this void by demonstrating how a computationally efficient 2D framework can be leveraged to achieve robust 3D segmentation results.

## Materials

### Datasets

To evaluate the proposed method, we perform experiments on three different well-documented segmentation datasets, as shown in Table 1. The datasets were chosen due to variations in imaging modalities, segmentation tasks, and clinical applications, thereby allowing for comprehensive analysis across a spectrum of medical imaging challenges. The Medical Segmentation Decathlon (MSD): Task01_BrainTumour (BRAIN_1) and MSD: Task04_Hippocampus (BRAIN_2) are two datasets from the Medical Segmentation Decathlon challenge [31], which was developed to test the generalisability of machine learning algorithms. The BRAIN_1 dataset includes multi-parametric magnetic resonance images mp-MRI from patients with either glioblastoma or lower-grade glioma. Ground-truth annotations of three tumour sub-regions (oedema, enhancing, and non-enhancing tumour) are provided. Collected from 19 different institutions, this dataset represents a subset of the data from the 2016 and 2017 Brain Tumor Segmentation (BraTS) challenges. The BRAIN_2 dataset consists of MRI images from healthy adults and adults with non-affective psychotic disorder, with annotations for the anterior and posterior of the hippocampus.

**Table 1:** Summary of biomedical datasets used in our experiment

Acronym	Description	Target	Modality	Volumes	Dimensions ($x$ pixels, $y$ pixels, $z$ slices, channels)
BRAIN_1	MSD: Task01 [31]	Brain tumor	mp-MRI	100	176x144x155x4
BRAIN_2	MSD: Task04 [31]	Hippocampus location	MRI	260	32x32x24-47x1
LIVER	LiTS 17 [32]	Liver and liver tumor	CECT	100	96x96x74-986x1

The Liver Tumor Segmentation Benchmark 2017 (LIVER) is a dataset which includes contrast-enhanced computed tomography CECT scans of the abdomen from various institutions [32]. Each scan is annotated with pixel-wise segmentation masks for the liver and tumours. It was introduced as part of the LiTS challenge to encourage the development of automatic liver lesion segmentation algorithms.

**Fig. 1:**
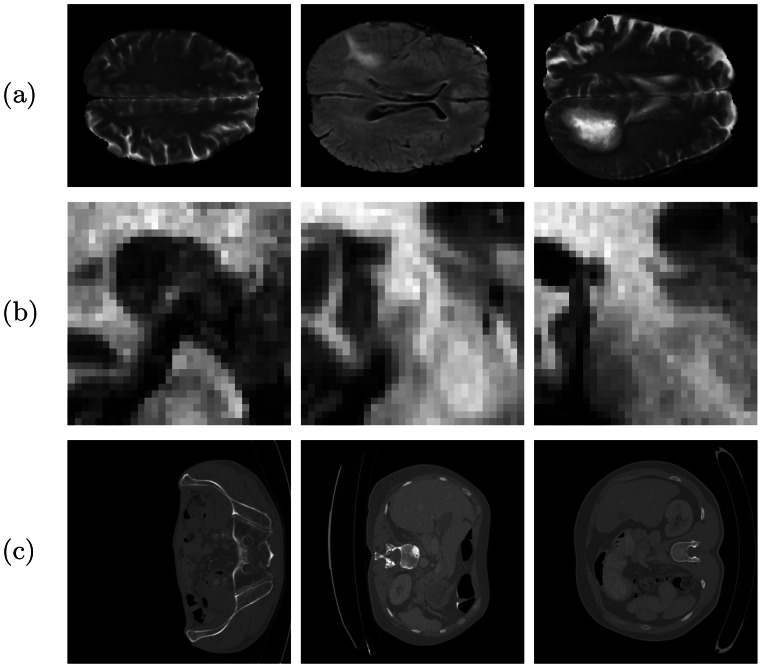
Sample 2D image slices from datasets: (**a**) BRAIN_1, (**b**) BRAIN_2, (**c**) LIVER

Some sample 2D slices for each dataset can be seen in Fig. 1.

#### Preprocessing

Due to computational resource limitations, preprocessing varied by dataset. All images were resized using bilinear interpolation and binary masks with nearest-neighbour interpolation. Cropping was used to remove excess background. The specific changes for each dataset were as follows. For the BRAIN_1 dataset, we applied a central crop of $176 \times 144$ pixels and used a random subset of 100 volumes. For the BRAIN_2 dataset, volumes were resized from an average of $50 \times 35$ pixels to $32 \times 32$ pixels. For the LIVER dataset, volumes were resized from $512 \times 512$ pixels to $96 \times 96$ pixels, and a random subset of 100 volumes was used. All datasets were randomly split into 70% training, 15% validation, and 15% test sets.

Data was standardised to a mean of 0 and a standard deviation of 1. For single-channel data, each 2D slice was standardised independently; for multi-channel data, each channel within each slice was standardised independently.

The annotations for each dataset were also converted into binary segmentation tasks. This modification reduces annotation complexity and improves training stability, particularly with imbalanced classes. For the BRAIN_1 dataset, we combined the classes (1) oedema, (2) non-enhancing tumour, and (3) enhancing tumour into a single ‘tumour’ class. For the BRAIN_2 dataset, we merged the (1) anterior and (2) posterior hippocampus classes into a single ‘hippocampus’ class. For the LIVER dataset, we combined the (1) liver and (2) tumour classes into a single ‘region-of-interest’ class. While these modifications alter the original challenge intended by the dataset curators, they allowed us to assess the performance of our approach under simplified conditions.

### Model architecture

While our proposed self-labelling method is model-agnostic, we implemented and tested it using a 2D U-Net. The network follows the original architecture defined by Ronneberger et al. [8], but with a reduced number of feature maps due to computational constraints; specifically, the number of convolutional filters starts at 8 in the first layer and doubles with each downsampling step to a maximum of 128 in the bottleneck.

To handle 3D and 4D datasets, 2D slices were extracted and processed independently. The same 2D U-Net architecture was used for all 2D model comparisons (the full 2D baseline, the central-slice-only model, and our self-labelling model) to ensure a fair comparison. The 3D baseline model used an analogous architecture but replacing 2D operations (e.g., convolutions, pooling) with 3D versions.

## Methods

### Incremental 2D self-labelling framework

To tackle the challenge of training segmentation models with minimal annotations, we propose a self-labelling framework for a 2D U-Net to iteratively generate labels and improve its performance on 3D medical volumes. The core problem addresses a scenario where only a single, central 2D slice of each 3D training image is annotated. Let $I_{\mathrm{train}} = \{I_n\}_{n=1}^N$ denote the training set of $N$ 3D images, where each volume $I_n$ has a depth of $z_n$. Let $M_{\mathrm{GT}} = \{M_n\}_{n=1}^N$ be the corresponding set of ground-truth annotations, where each $M_n$ is the mask for the central slice of volume $I_n$. All slice indexing is zero-based; for a volume with $z_n$ slices, the central slice is at index $\lfloor z_n / 2 \rfloor$.

**Algorithm 1:**
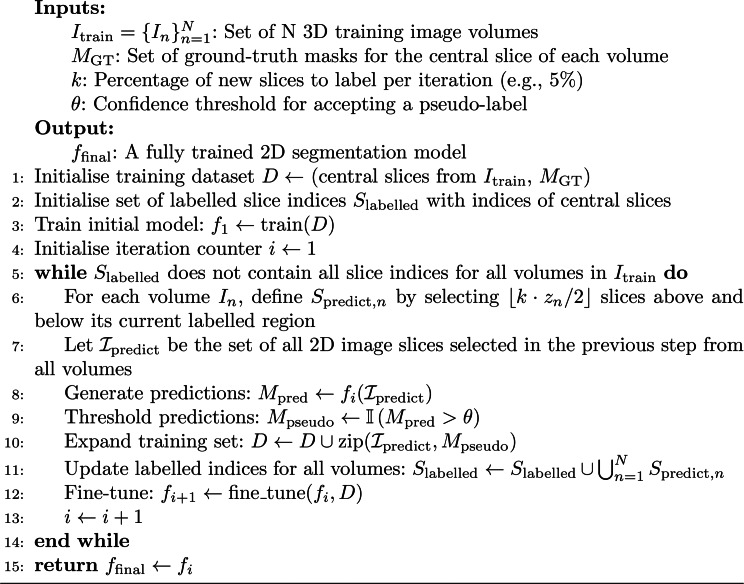
Incremental 2D self-labelling for 3D volume segmentation

**Fig. 2:**
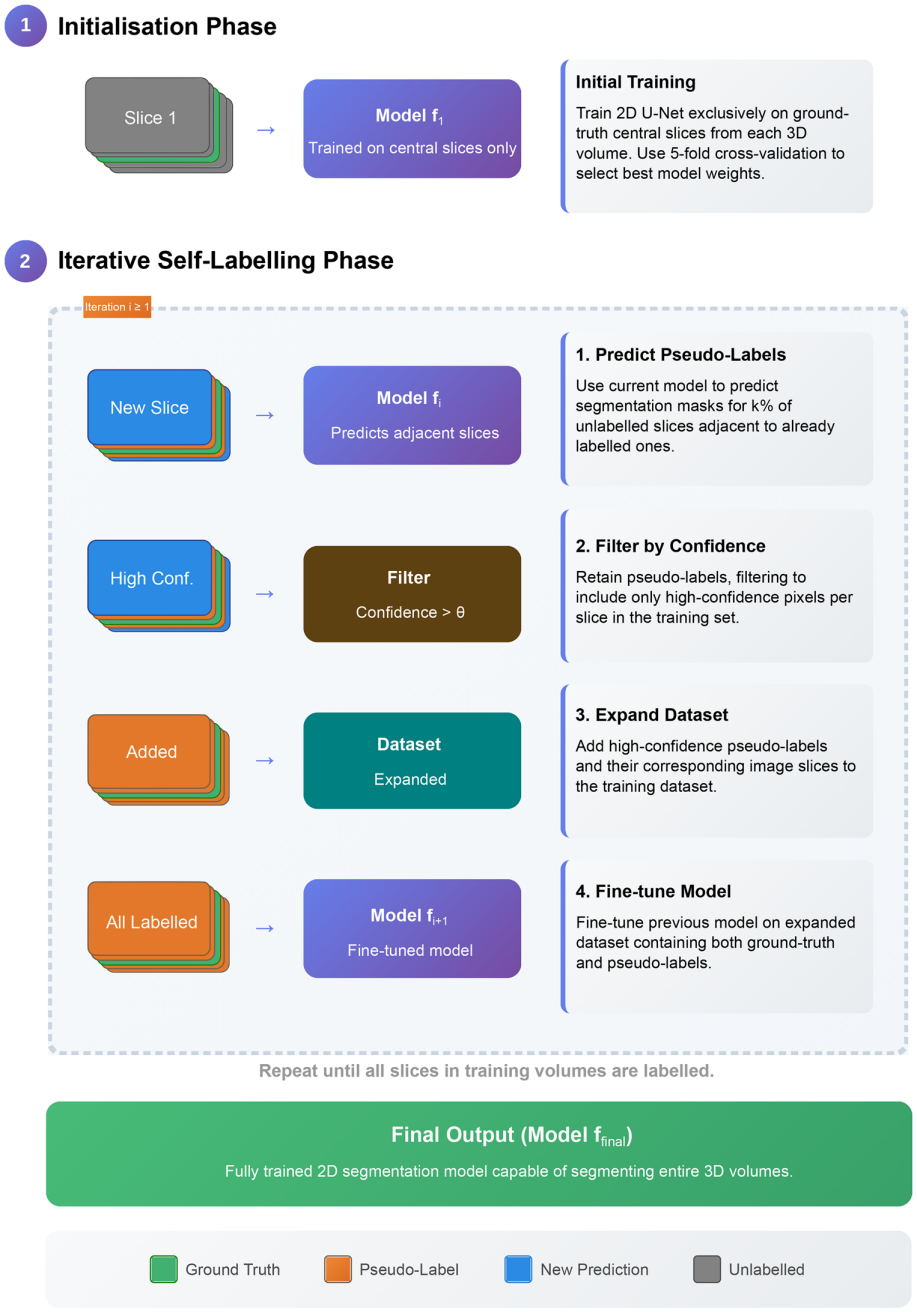
Overview of the proposed incremental 2D self-labelling framework. The process has two main phases. (1) initialisation: a baseline model $f_1$ is trained using only the central slice annotations from each 3D volume. (2) iterative self-labelling: In each iteration $i$, the current model $f_i$ predicts segmentation masks for neighbouring unlabelled slices. These predictions are thresholded to produce binary pseudo-labels, added to the training set, and used to fine-tune the model to obtain $f_{i+1}$. This process repeats until all slices are labelled, resulting in the final model $f_{\mathrm{final}}$

The framework, detailed in Algorithm 1, consists of an initialisation phase followed by an iterative self-labelling and fine-tuning cycle. The entire process is also illustrated in Fig. 2.

#### Step 1: Initialisation

The process begins by training an initial 2D U-Net model exclusively on the sparsely annotated dataset of central slices. To ensure robustness and select the best starting point, we employ a 5-fold cross-validation process and use the model weights that yield the highest Dice Similarity Coefficient (*DSC*) on the validation data. This initial phase enables the model $f_1$ to learn the most representative features of the target structures, which are often best captured in the central part of a medical volume. Although the datasets used in this study have full 3D annotations, our method simulates a real-world scenario where only these central slices would be manually annotated.

#### Step 2: Iterative self-labelling and fine-tuning

Following initialisation, the model enters an iterative loop detailed in Algorithm 1. In each iteration, the model $f_i$ predicts pseudo-labels for a new set of adjacent, unlabelled slices. The number of slices to add is determined by the hyperparameter $k$ (5% of each volume’s depth, $z_n$, in our experiments as determined by parameter analysis). These are selected symmetrically above and below the currently labelled region. The predictions are filtered using a confidence threshold $\theta$ to create partial masks, which are then added to the training set. The model is then fine-tuned on this expanded dataset to produce $f_{i+1}$. This process expands the labelled region outwards from the central slice until the entire volume is pseudo-labelled.

This cycle continues until a complete pseudo-label mask has been generated for every volume in the training set. The process terminates when the depth of each generated 3D pseudo-label volume matches the depth of its corresponding source image. It is important to note that slices are not skipped due to low confidence; if all pixels in a predicted slice fall below the confidence threshold $\theta$, the slice is treated as a background-only mask and is still added to the training set for the next fine-tuning iteration.

**Fig. 3:**
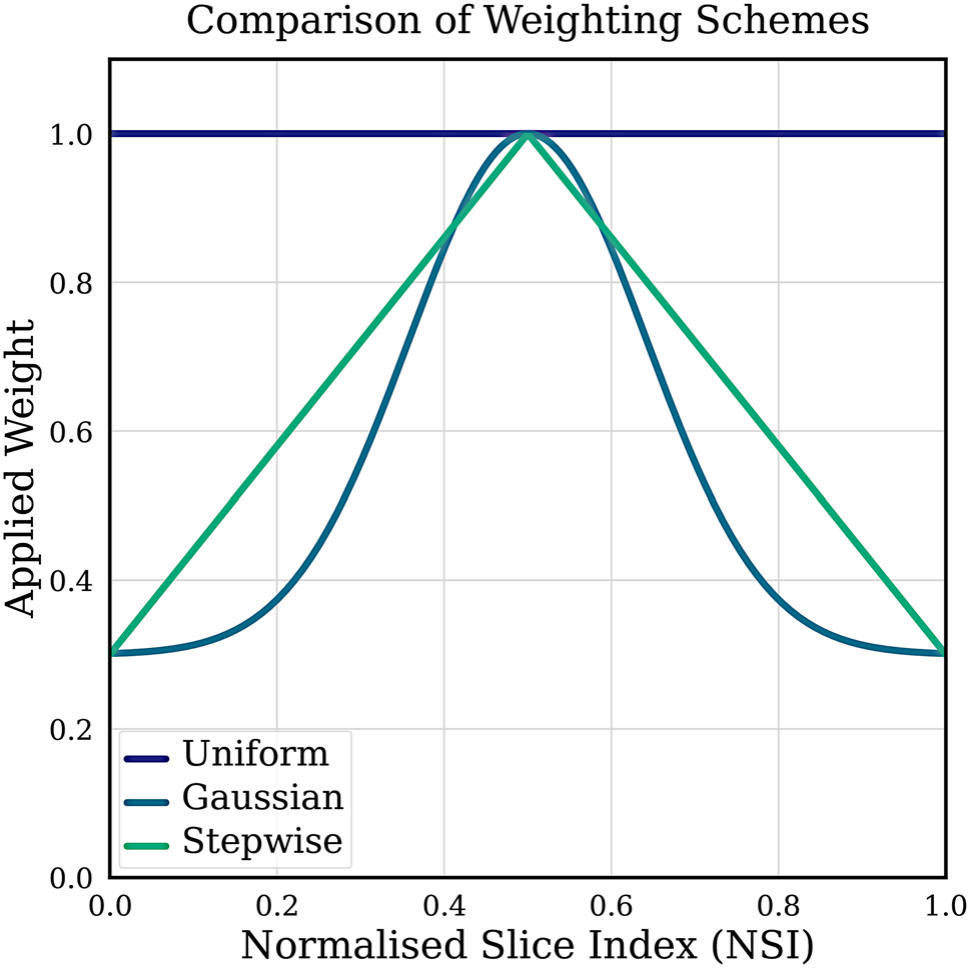
A visual comparison of the three weighting schemes investigated: uniform, Gaussian, and stepwise. The weight applied to each pseudo-label is shown as a function of the slice’s normalised position within the volume (from 0 at the first slice to 1 at the last slice)

As pseudo-labels for slices further from the central ground-truth slice are expected to be less accurate, we explored applying different weighting schemes to the loss function during training. To standardise slice locations across datasets with varying depths, we define a Normalised Slice Index NSI. The NSI ranges from 0 (first slice) to 1 (last slice), with the central ground-truth slice positioned at an NSI of 0.5. We investigated three potential schemes (Uniform, Gaussian, and Stepwise), visualised in Fig. 3. The Uniform scheme weights all labels equally. The Gaussian and Stepwise schemes reduce the weight of slices far from the central slice. However, the final model presented in this study utilises the Uniform scheme (a weight of 1.0 for all slices), as our sensitivity analysis revealed that the more complex Gaussian and Stepwise schemes did not yield a significant performance improvement.

### Parameter sensitivity analysis

We performed a sensitivity analysis on the BRAIN_1 dataset to evaluate how different hyperparameters affect the performance of the model. We investigated the impact of: (1) the confidence threshold $\theta$ for pseudo-label filtering; (2) the percentage of new slices added per iteration with respect to the total volume depth, $k$; (3) the three proposed loss function weighting schemes (Uniform, Gaussian, and Stepwise); and (4) the amount of initial ground-truth data used for training.

### 3D boundary fidelity and smoothness analysis

To assess the boundary accuracy and fragmentation of the final 3D segmented volumes, a post-hoc analysis was performed on the test set predictions against their corresponding ground truth masks. For this, we calculated the HD95, a metric robust to small outliers that evaluates the distance between two surfaces and is widely used in evaluating medical image segmentation methods [33].

The symmetric HD between two point sets $A$ and $B$ is defined as the maximum of the directed distances between them. Let $\partial M_{\mathrm{pred}}$ be the set of surface voxels of a predicted volume and $\partial M_{\mathrm{GT}}$ be the surface voxels of its ground truth. The distance from a point $p$ to a set $S$ is $d(p, S) = \min_{s \in S} \| p - s \|$. The set of all surface distances is the collection of minimum distances from every point on one surface to the other, and vice-versa:

1 $$\mathcal{D} = \{d(p, \partial M_{\mathrm{GT}}) \mid p \in \partial M_{\mathrm{pred}} \} \cup \{d(q, \partial M_{\mathrm{pred}}) \mid q \in \partial M_{\mathrm{GT}} \}$$

The HD95 is the 95th percentile of this combined set of distances $\mathcal{D}$. Using the 95th percentile mitigates the impact of a few outlier voxel predictions, providing a more stable and clinically relevant measure of boundary deviation. A lower HD95 value indicates that the predicted segmentation boundary is a less fragmented prediction with boundaries that are more accurately aligned with the ground truth. This is clinically relevant, as accurate and continuous predictions are essential for reliable downstream analysis.

### Training details

The experiments were conducted using the TensorFlow v2.10.1 framework, with distributed training across an NVIDIA RTX 4070 and RTX 3060 Ti, providing a combined 20 GB of VRAM. For our self-labelling approach, the initial model was trained exclusively on the central ground-truth slices using 5-fold cross-validation. Each fine-tuning round in our iterative framework consisted of training for up to 150 epochs using the RMSprop optimiser with an initial learning rate of $1 \times 10^{-3}$. The learning rate was reduced by a factor of 0.1 if the validation loss plateaued, and training was subject to an early stopping condition with a patience of 6 epochs. The weights from the epoch with the best validation loss were saved and used as the starting point for the next iteration.

Mini-batches are formed by pooling all available 2D slices (both ground-truth and pseudo-labelled) from all 3D volumes in the training set. The data generator then creates batches by randomly sampling from this combined pool of 2D slices. This ensures that each batch contains a diverse mix of examples from different subjects and slice locations.

Basic image augmentation was applied exclusively during the initial training of model $f_1$ on ground-truth central slices. Preliminary experiments showed that applying augmentation during the fine-tuning steps on pseudo-labelled slices resulted in reduced model performance, therefore were not applied on pseudo-labels. This is likely due to because pseudo-labels containing less certain boundaries and inherent noise from the model’s own predictions, and aggressive geometric augmentation may exacerbate this noise, forcing the model to learn incorrect or noisy spatial features. Augmentations included horizontal and vertical flips, and random rotations up to $90^\circ$.

Training used the binary cross-entropy (BCE) loss, defined below, which measures the pixel-wise discrepancy between predicted probabilities and ground-truth labels:

2 $$\mathrm{BCE} = -\frac{1}{P} \sum_{j=1}^{P} \left[ y_j \cdot \log(p_j) + (1 - y_j) \cdot \log(1 - p_j) \right],$$

where $P$ is the total number of pixels in a batch, $y_j$ is the ground-truth label for pixel $j$ (either 1 or 0), and $p_j$ is the predicted probability of pixel $j$ belonging to the positive class. The final model used no slice-level weighting.

## Results

### Performance metrics

To measure the performance of our models, we use a combination of common segmentation metrics, including *DSC*, *IoU*, True Negative Rate TNR and True Positive Rate TPR, as shown in Eqs. 3, 4, 5 and 6, respectively.

3 $$ DSC = \frac{{2{TP}}}{{2TP+FP+FN}},$$

4 $$ IoU = \frac{TP}{TP+FP+FN},$$

5 $$TNR = \frac{TN}{TN+FP},$$

6 $$TPR = \frac{TP}{TP+FN},$$

where TP, TN, FP and FN refer to true positive, true negative, false positive and false negative regions, respectively. Both the DSC and IoU, with a range of 0 to 1, assess the overlap between sets. However, unlike the DSC, the IoU penalises under- and over-segmentation more than the DSC.

### Quantitative evaluation

In Table 2, we present the quantitative results for five different methods in the three datasets used in this study, with the 3D baseline and 2D baseline models trained on full ground-truth annotations for benchmark comparisons. To evaluate the effectiveness of our approach, we also provide the results of a 2D self-labelling U-Net which follows the same method previously detailed but instead randomly predicts data to then use for training/validation. The number of pseudo-labels incorporated into the dataset between iterations for both approaches is kept constant between both ($k=5\%$). The gradual expansion of 2D self-labelling consistently outperformed the 2D central slices only method across all datasets, where the greatest performance gains were on both the BRAIN_1 and BRAIN_2 datasets, with DSC and IoU increasing by an average of 15.95% and 26.75% between the 2D central slices only model, and 2D self-labelling model. Although the 2D self-labelling model did not outperform benchmarks with fully annotated ground-truth data, its competitive performance suggests its potential utility in scenarios with minimal annotated data. The 2D self-labelling (random expansion) model also showed improvements over the 2D central slices only model, which supports the efficacy of using pseudo-labels to improve performance; however, it did not achieve as good performance as the gradual expansion method.

**Table 2:** Performance metrics for different models on the different datasets. The best-performing metrics for non-benchmark models are highlighted in bold

Model type	Metric	Dataset		
		BRAIN_1	BRAIN_2	LIVER
3D baseline	DSC	0.859$\pm$0.011	0.882$\pm$0.006	0.896$\pm$0.008
	IoU	0.658$\pm$0.008	0.748$\pm$0.000	0.667$\pm$0.006
	TPR	0.823$\pm$0.007	0.891$\pm$0.001	0.836$\pm$0.000
	TNR	0.998$\pm$0.001	0.996$\pm$0.000	0.999$\pm$0.000
2D baseline	DSC	0.831$\pm$ 0.002	0.812$\pm$0.003	0.851$\pm$0.007
	IoU	0.578$\pm$ 0.002	0.639$\pm$0.003	0.682$\pm$0.009
	TPR	0.805$\pm$ 0.007	0.782$\pm$0.004	0.798$\pm$0.015
	TNR	0.998$\pm$ 0.000	0.992$\pm$0.000	0.998$\pm$0.000
2D central	DSC	0.476$\pm$0.011	0.484$\pm$0.002	0.667$\pm$0.007
Slices only	IoU	0.215$\pm$0.004	0.292$\pm$0.002	0.178$\pm$0.004
	TPR	**0.892**$\pm$**0.005**	0.443$\pm$0.001	0.666$\pm$0.013
	TNR	0.960$\pm$0.001	0.979$\pm$0.002	**0.992**$\pm$**0.001**
2D self-labelling	DSC	**0.664**$\pm$**0.010**	**0.615**$\pm$**0.003**	**0.675**$\pm$**0.012**
	IoU	**0.621**$\pm$**0.008**	0.421$\pm$0.003	**0.506**$\pm$**0.020**
	TPR	0.568$\pm$0.003	**0.578**$\pm$**0.003**	**0.670**$\pm$**0.024**
	TNR	**0.999**$\pm$**0.000**	**0.984**$\pm$**0.000**	**0.992**$\pm$**0.001**
2D self-labelling	DSC	0.593$\pm$0.011	0.564$\pm$0.002	0.473$\pm$0.015
(random expansion)	IoU	0.480$\pm$0.006	**0.438**$\pm$**0.003**	0.433$\pm$0.019
	TPR	0.620$\pm$0.005	0.570$\pm$0.002	0.665$\pm$0.023
	TNR	0.968$\pm$0.001	0.975$\pm$0.001	0.992$\pm$0.001

**Fig. 4:**
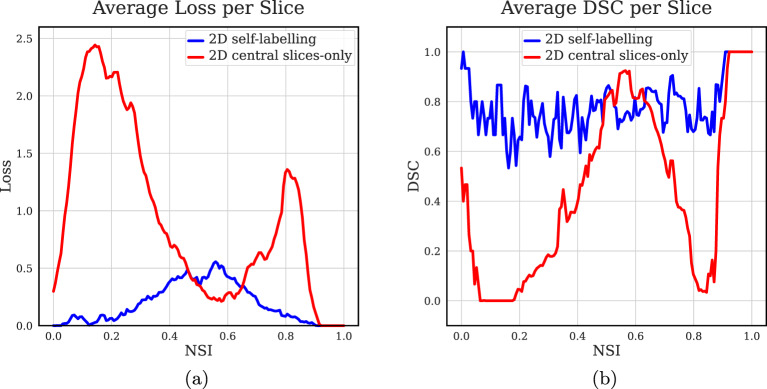
2D self-labelling and 2D central slices only models applied to BRAIN_1 test data, with (**a**) loss and (**b**) DSC averaged across all slices

Figure 4 shows the performance comparison between the 2D self-labelling, and the 2D central slices only models averaged across all 2D slices of the BRAIN_1 test data. As seen in Fig. 4a, the 2D self-labelling model has on average reduced loss throughout the volume. This is further validated in Fig. 4b, where average DSC is improved across the entire volume. However, it has a higher average loss and reduced average DSC closer to the central slice at an NSI of approximately 0.5 to 0.6.

### 3D continuity analysis

The 3D boundary accuracy analysis for the BRAIN_1 dataset is summarised in Table 3. The fully supervised 3D baseline established the strongest performance with the lowest mean HD95 of 11.57 mm, indicating the most accurate boundary alignment to the ground-truth annotations.

**Table 3:** 3D boundary fidelity and smoothness analysis for the BRAIN_1 dataset, evaluated using HD95. Lower values indicate better performance. The proposed model is indicated in bold

Model Type	Mean HD95 $\pm$ Std. Dev. (mm)
3D baseline	11.57 $\pm$ 13.29
2D baseline	19.00 $\pm$ 23.12
2D central slices only	69.88 $\pm$ 19.54
**2D self-labelling**	**36.46** $\boldsymbol{\pm}$ **32.54**

In contrast, the 2D central slices only model was the least accurate, producing a mean HD95 of 69.88 mm, which highlights a significant deviation from the ground truth boundaries when trained on minimal data. The proposed 2D self-labelling model achieved a mean HD95 of 36.46 mm. While this did not surpass the fully supervised 2D baseline (19.00 mm), it demonstrates a substantial improvement, reducing the boundary error by nearly half compared to the 2D central slices only model. This finding shows that on average the self-labelling framework successfully improves the fragmentation and spatial accuracy of the final 3D prediction by propagating knowledge from the single annotated slice.

### Parameter sensitivity analysis

**Fig. 5:**
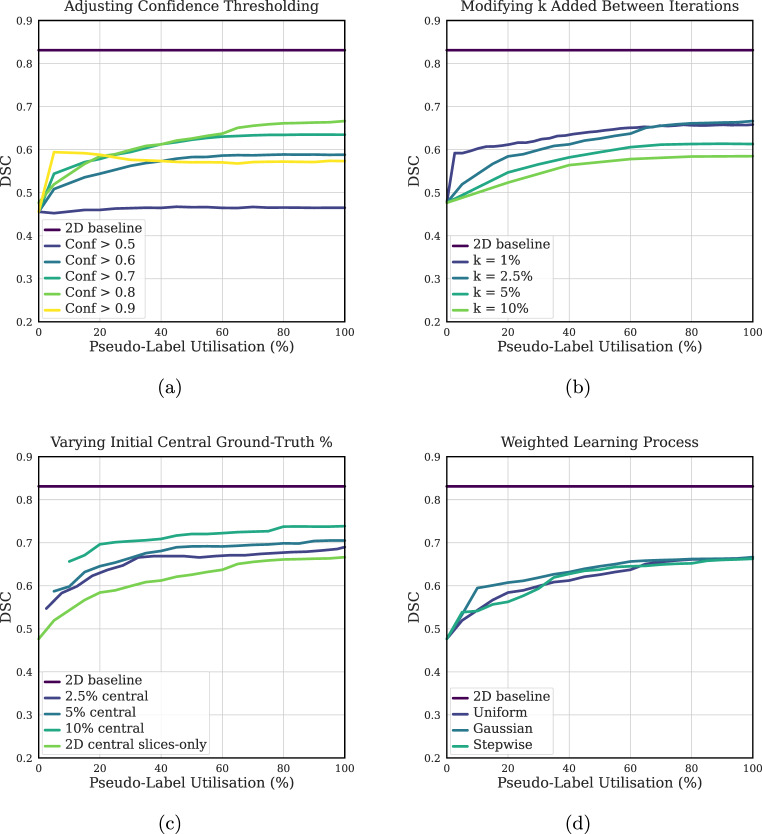
Parameter sensitivity analysis for the pseudo-labeling approach on the BRAIN_1 dataset. Each plot shows the model’s performance (DSC) on the test set as a function of the amount of pseudo-labelled data used in training. The 2D baseline, trained on all ground-truth volumes on a slice-by-slice basis, is included for reference. The parameters varied in each subplot are: (**a**) the confidence threshold for generating pseudo-labels; (**b**) the percentage of pseudo-labels added per iteration; (**c**) the size of the initial ground-truth training set; and (**d**) the weighting strategy applied to the pseudo-labels

Our parameter sensitivity analysis on the BRAIN_1 dataset revealed that adjusting the hyperparameters had a substantial impact on the resulting model performance. The confidence threshold for pseudo-label filtering was found to be optimal between 0.6 and 0.8 (Fig. 5a). While using high-confidence predictions helps minimise error propagation, an excessively high threshold of 0.9 reduced the final test DSC, likely due to the exclusion of too much useful information. Similarly, the amount of pseudo-labels added between each iteration, $k$, was optimal at 5% of the volume (Fig. 5b). A larger step size of 10% decreased the final test DSC by 5.1%, suggesting that a more gradual expansion is beneficial.

**Fig. 6:**
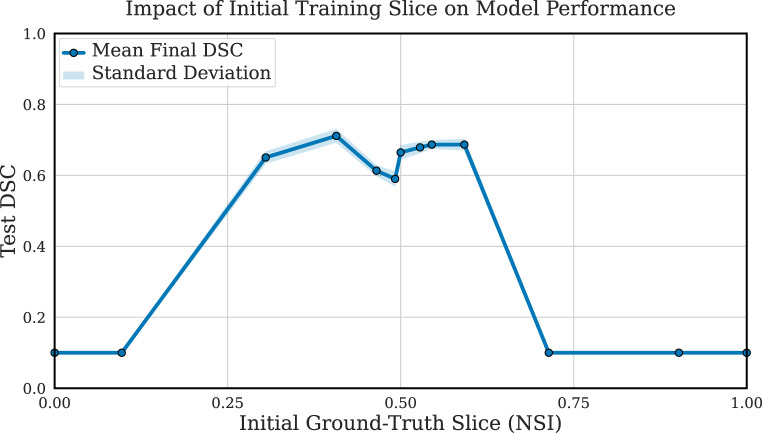
Final test DSC of the 2D self-labelling model when initial training is performed using different single-slice ground-truth annotations from varying positions within the training volumes on the BRAIN_1 dataset. Results show that starting from slices near the centre of the volume (NSI ≈ 0.3-0.7) yields better performance, while peripheral slices lead to significantly worse outcomes

The initial conditions for the training process were also found to be critical. As indicated by Fig. 6, commencing the initial model with ground-truth slices from the feature-rich central third of the volume proved effective. Initiating with a slice outside this region resulted in a notably poor final test performance, mainly due to the limited annotated area for the initial model to learn from. Furthermore, increasing the amount of initial ground-truth data yielded notable and persistent performance improvements throughout the self-labelling process (Fig. 5c). In contrast, all weighted-learning approaches yielded similar performance to the standard unweighted method, with less than a 1% variance in the final test DSC (Fig. 5d).

Table 4 shows how different methods compare computational costs. For the 3D baseline, 2D baseline, and 2D central slices only, the times provided exclude 5 cross-fold validations, only showing the average time across all 5 splits. As expected, the 2D central slices only model is the quickest to train but performs poorly. Both 3D and 2D models take roughly the same time to train. However, the 2D self-labelling model takes the longest to train due to repeated cycles on augmented datasets, with an average training time 35.37 times greater than the 2D central slices only model.

**Table 4:** Model parameters and training time

Model Type	Dataset	# Parameters	Training Time (minutes)
3D baseline	BRAIN_1	1,412,417	57.49
	BRAIN_2	1,411,769	1.88
	LIVER	1,411,769	226.15
2D baseline	BRAIN_1	485,889	46.16
	BRAIN_2	485,673	2.58
	LIVER	485,673	224.01
2D central slices only	BRAIN_1	485,889	6.47
	BRAIN_2	485,673	1.25
	LIVER	485,673	43.87
2D self-labelling	BRAIN_1	485,889	336.72
	BRAIN_2	485,673	38.25
	LIVER	485,673	1444.05
2D self-labelling (random expansion)	BRAIN_1	485,889	346.81
	BRAIN_2	485,673	37.12
	LIVER	485,673	1421.64

### Qualitative evaluation

**Fig. 7:**
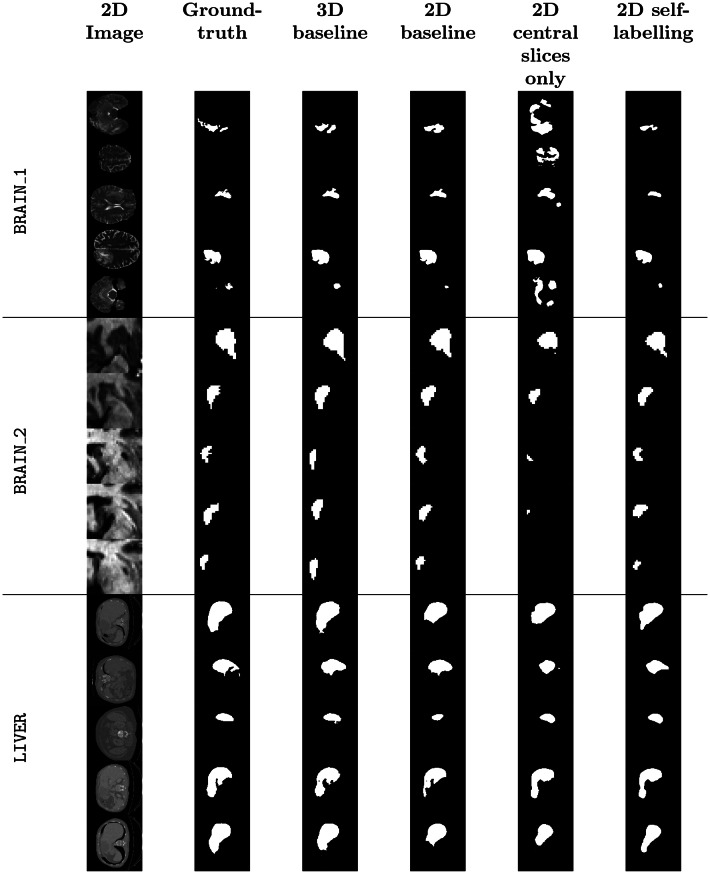
Qualitative comparative analysis of the segmentation performance. The source 2D image slice obtained from test volumes is shown alongside the ground-truth mask and subsequent predictions from each model type are detailed

Figure 7 shows randomly selected segmentations from the test data of each dataset. Predictions from each model type are presented alongside the ground-truth. These images, drawn from thousands of slices, are intended solely for illustration and may not fully reflect overall segmentation performance. Nonetheless, some qualitative differences between the 2D central-slice and 2D self-labelling models align with the quantitative results in Table 2.

For the BRAIN_1 dataset, the self-labelling model substantially reduces over-segmentation, particularly on slices farther from the centre that were not included in the training data. In the BRAIN_2 dataset, predicted boundaries and areas are closer to the ground-truth, consistent with a 13.5% improvement in TPR. Improvements on the LIVER dataset are less pronounced, likely due to the dataset’s large size and complexity, with volumes ranging from $z \in [74, 986]$. In some cases, using a single annotated slice represents only 0.001% of the total volume.

**Fig. 8:**
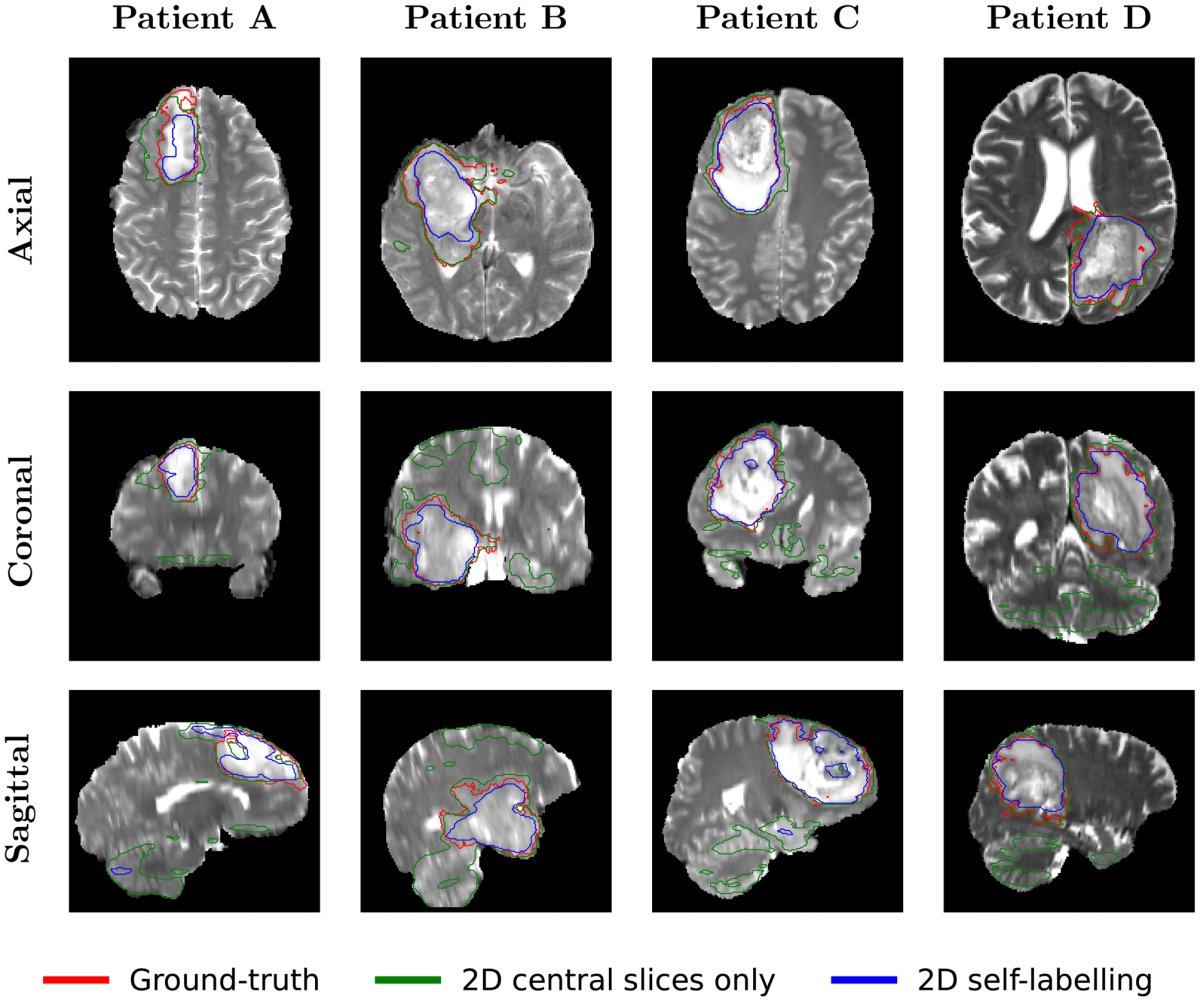
Qualitative segmentation results for four representative cases (**A-D**) from the BRAIN_1 test dataset. Each row corresponds to a specific anatomical view (Axial, Coronal, Sagittal). The coloured contours represent the ground-truth (red), 2D central slices only model (green), and 2D self-labelling model (blue)

To provide a more comprehensive volumetric assessment, Fig. 8 presents orthogonal views of a representative BRAIN_1 segmentation. While Fig. 7 shows 2D slice performance, this cross-plane visualisation highlights the full 3D effect of our method. The baseline model trained only on central slices produces fragmented, incomplete predictions, with disjoint segments visible in the coronal and sagittal planes. In contrast, our incremental self-labelling approach generates a coherent 3D volume that closely matches the ground truth. This qualitative improvement corresponds to a significant reduction in mean HD95 from 69.88 mm to 36.46 mm (Table 3), demonstrating the framework’s ability to enforce 3D consistency from a single annotated slice.

## Discussion

Our approach addresses a complementary setting to mainstream semi-supervised learning, which typically assumes many unlabelled volumes and some fully annotated ones. By contrast, we focus on propagating labels from a single annotated slice, and therefore our evaluation emphasises baselines tailored to this sparse-annotation problem rather than direct competition with general semi-supervised methods.

This work demonstrates that a simple incremental pseudo-labelling approach can improve segmentation performance under severe annotation constraints. The quantitative results demonstrate the effectiveness of the self-labelling strategy. Incrementally incorporating pseudo-labels alongside ground-truth data enables the model to learn relevant features in neighbouring slices, resulting in improved performance on unseen data. Analysis of loss and DSC across the entire volume shows consistent gains beyond the central slices, highlighting the benefit of exposing the model to a broader range of image contexts.

A key finding is the impact on 3D continuity, evidenced by the reduction in mean HD95 from 69.88 mm for the 2D central slices only model to 36.46 mm with our method. This shows successful propagation of spatial knowledge from a single annotated slice, allowing the model to learn a more continuous and morphologically plausible 3D structure.

The parameter sensitivity results highlight important considerations for designing an effective self-labelling pipeline. (i) Selecting a pixel confidence threshold in the 0.6–0.8 range strikes a practical balance: high enough to filter unreliable pseudo-labels, but not so strict that it excludes valuable training signals. (ii) Gradually introducing pseudo-labels between iterations, rather than in large steps, improves performance by maintaining stability and limiting the impact of early misclassifications. (iii) The quality and location of initial ground-truth slices are critical—central slices rich in features allow the model to extract more useful representations, improving downstream pseudo-labelling. (iv) Increasing the number of initial annotations consistently strengthens performance, reinforcing the value of a robust starting point in low-data regimes. These findings collectively underline that while the method is designed for minimal supervision, careful tuning of initial conditions and expansion strategy is essential to exploit its full benefit.

The similarity in performance among weighted-learning approaches likely results from slices farther from the centre containing mostly background pixels in the BRAIN_1 dataset. Consequently, reducing the impact of pseudo-labels on these outer slices has minimal effect, as informative content is concentrated near the centre on this dataset.

Efficiency comparisons show the self-labelling method requires longer training but offers improved performance without additional manual annotations, making it viable when time permits. Compared to the 3D benchmark, the 2D models use roughly one-third of the parameters, underscoring their computational efficiency.

### Limitations

Our approach has several limitations that warrant consideration. The self-labelling model is prone to propagating errors, as it cannot reliably correct mistaken pseudo-labels once introduced. Although high-confidence thresholds mitigate this, early errors may still compound [34].

Being a 2D framework, the model lacks volumetric context. While computationally efficient, this limits its ability to capture depth-dependent features that can improve segmentation performance [35].

Structural continuity across neighbouring slices is assumed, which may not hold in anatomically heterogeneous datasets (e.g., abdominal CT or whole-body PET/CT) or in modalities with low through-plane resolution or sparse slice sampling. Effectiveness may degrade when foreground structures are sparse or spatially disjoint.

The approach also assumes that the target object appears, at least partially, in the initially annotated slices. Small or peripheral structures (e.g., small tumours) may be absent in central slices, leading to poor or absent pseudo-label propagation.

Segmentation of anisotropic structures aligned with the slice axis, such as tubular objects (e.g., the aorta or spinal cord), may be less reliable, as individual slices may poorly represent these features.

Experiments were performed on downsampled images due to computational constraints. While this allowed for a thorough evaluation of the framework’s logic, performance on full-resolution (e.g., 512x512) images, where finer contextual details are present, remains to be explored.

The choice of initial annotated slices is critical. If these do not adequately represent the target anatomy, subsequent pseudo-labels may be unreliable, limiting robustness in low-data or highly variable settings.

Based on these limitations, the framework is most suitable for segmenting anatomies that (i) span multiple contiguous slices, (ii) have substantial representation in central regions of the scan, and (iii) are not highly anisotropic along the slice axis. Applications involving small, sparse, or peripherally located targets may require modified sampling strategies or volumetric models.

### Future work

Several extensions could enhance the robustness and generalisability of the method. Improved initialisation using multiple annotated slices across the volume, rather than a single central slice, may provide broader contextual coverage and reduce sensitivity to initial slice placement.

Adaptive expansion strategies, such as guiding pseudo-label propagation using model uncertainty, anatomical boundaries, or confidence maps, could prove more effective than fixed slice-wise progression, particularly in datasets with complex or variable spatial structure.

To improve label reliability and mitigate confirmation bias during iterative training, future work could incorporate teacher-student frameworks, soft pseudo-labels, or consistency regularisation. These mechanisms can help prevent the model from reinforcing its own errors, especially in low-confidence regions. Additional safeguards, including adaptive confidence thresholds or uncertainty-guided correction, may further reduce error accumulation over time.

Finally, incorporating shallow 3D or 2.5D architectures may provide a better balance between spatial context and computational efficiency. Similarly, applying the 2D framework across multiple anatomical planes (axial, coronal, and sagittal) and fusing the resulting predictions could yield more isotropic and robust 3D segmentations, particularly for anisotropic structures. Automated selection of initial annotated slices based on image content or model uncertainty could further reduce reliance on manual expertise and improve early pseudo-label quality.

## Conclusion

This study presented a simple yet effective incremental pseudo-labelling approach to improve 3D segmentation performance using 2D models under severe annotation constraints. Evaluations across multiple datasets show that starting from a single central slice, the method can produce accurate and morphologically consistent segmentation across entire volumes. Gradual exposure to neighbouring slices enhances both standard performance metrics and 3D continuity, despite the limited supervision. While the method performs best on centrally located, isotropic structures, it offers a practical and efficient solution in low-data regimes. Key design factors, such as confidence thresholds, annotation placement, and expansion strategy, are critical to its success. Future work will explore extensions to multi-class segmentation, adaptive propagation strategies, and hybrid 2D–3D architectures to improve robustness across more complex anatomical settings.

## Data Availability

The code for this paper is available at: https://github.com/muanderson/Incremental2D-SelfLabel3D. The MSD: Task01_BrainTumour [31] and MSD: Task04_Hippocampus [31] datasets are available at https://medicaldecathlon.com/. The LiTS 17 [32] dataset is available at https://academictorrents.com/.

## Abbreviations

AI: Artificial intelligence
mm: Millimetres
mp-MRI: Multi-Parametric magnetic resonance images
CECT: Contrast-Enhanced computed tomography
CT: Computed tomography
NSI: Normalised slice index
HD95: 95th percentile hausdorff distance
HD: Hausdorff distance
DSC: Dice similarity coefficient
IoU: Intersection over Union
TNR: True negative rate
TPR: True positive rate
