# Correction to: Incremental 2D self-labelling for effective 3D medical volume segmentation with minimal annotations

**DOI:** 10.1186/s12880-026-02270-x

**Published:** 2026-03-16

**Authors:** Matthew Anderson, Maged Habib, David H. Steel, Boguslaw Obara

**Affiliations:** 1 School of Computing, Newcastle University, 1, Urban Sciences Building, Science Square, 1 Science Square, Newcastle Upon Tyne, NE4 5TG UK; 2 Sunderland Eye Infirmary, National Health Service, Queen Alexandra Rd, Sunderland, SR2 9HP UK; 3 Biosciences Institute, Newcastle University, Catherine Cookson Buildinge, Newcastle Upon Tyne, NE2 4HH UK

**Correction to: Anderson et al. BMC Medical Imaging (2025) 25:450.**

10.1186/s12880-025-01991-9.

Following the publication of the original article, the author reported two errors.

- In Fig. 3, the letter “S” in the x‑label appears in a different color in the published version.
- In Fig. 6, the published caption contains “(NSI ≈ 0.3{0.7)”, which is incorrect. The correct caption should be:

“Final test DSC of the 2D self-labelling model when initial training is performed using different single-slice ground-truth annotations from varying positions within the training volumes on the BRAIN_1 dataset. Results show that starting from slices near the centre of the volume (NSI ≈ 0.3–0.7) yields better performance, while peripheral slices lead to significantly worse outcomes.”

The original article has been corrected.

